# Common Commercial and Consumer Products Contain Activators of the Aryl Hydrocarbon (Dioxin) Receptor

**DOI:** 10.1371/journal.pone.0056860

**Published:** 2013-02-18

**Authors:** Bin Zhao, Jessica E. S. Bohonowych, Alicia Timme-Laragy, Dawoon Jung, Alessandra A. Affatato, Robert H. Rice, Richard T. Di Giulio, Michael S. Denison

**Affiliations:** 1 State Key Laboratory of Environmental Chemistry and Ecotoxicology, Research Center for Eco-Environmental Sciences, Chinese Academy of Sciences, Beijing, China; 2 Department of Environmental Toxicology, University of California Davis, Davis, California, United States of America; 3 Nicholas School of the Environment, Duke University, Durham, North Carolina, United States of America; University of Cincinnati, United States of America

## Abstract

Activation of the Ah receptor (AhR) by halogenated aromatic hydrocarbons (HAHs), such as 2,3,7,8-tetrachlorodibenzo-p-dioxin (TCDD, dioxin), can produce a wide variety of toxic and biological effects. While recent studies have shown that the AhR can bind and be activated by structurally diverse chemicals, how widespread of these AhR agonists are in environmental, biological and synthetic materials remains to be determined. Using AhR-based assays, we demonstrate the presence of potent AhR agonists in a variety of common commercial and consumer items. Solvent extracts of paper, rubber and plastic products contain chemicals that can bind to and stimulate AhR DNA binding and/or AhR-dependent gene expression in hepatic cytosol, cultured cell lines, human epidermis and zebrafish embryos. In contrast to TCDD and other persistent dioxin-like HAHs, activation of AhR-dependent gene expression by these extracts was transient, suggesting that the agonists are metabolically labile. Solvent extracts of rubber products produce AhR-dependent developmental toxicity in zebrafish in vivo, and inhibition of expression of the metabolic enzyme CYP1A, significantly increased their toxic potency. Although the identity of the responsible AhR-active chemicals and their toxicological impact remain to be determined, our data demonstrate that AhR active chemicals are widely distributed in everyday products.

## Introduction

2,3,7,8-Tetrachlorodibenzo-p-dioxin (TCDD, dioxin) and related dioxin-like chemicals are widespread environmental contaminants that produce a variety of toxic and biological effects, most of which are mediated by the aryl hydrocarbon (dioxin) receptor (AhR), a ligand-dependent nuclear receptor [1]–[5]. Although most TCDD-like AhR agonists are structurally related, recent studies suggest a high degree of promiscuity in the ligand binding specificity of the AhR [6]–[9]. During our analysis of solvent extracts of food products for AhR agonists [10], the inadvertent use of a rubber cap liner instead of a Teflon cap liner on vials containing the extracting solvent (DMSO) revealed that chemicals readily extracted from the rubber cap liner could maximally activate AhR DNA binding; no activation was observed with DMSO stored in Teflon-capped vials. These results, coupled with our recent identification of AhR agonists in extracts of commercial newspapers [11], [12] and automobile tires [13], prompted the present investigation to determine how widely distributed AhR-active chemicals are in common commercial and consumer products (rubber, plastic, paper, etc.). Given the documented ability of the AhR to respond to a wide range of exogenous and endogenous chemicals, the present work not only contributes to our understanding of the diversity and widespread nature of AhR agonists, but identifies putative sources of AhR ligands that can complicate experimental studies of AhR signal transduction.

## Materials and Methods

### Chemicals and extractions

TCDD and [^3^H]TCDD (37 Ci/mmol) were from S. Safe (Texas A&M University, College Station, TX), 2,3,7,8-tetrachlorodibenzofuran (TCDF) from Accustandard (New Haven, CT), [^32^P]ATP (6000 Ci/mmol) from Amersham (Arlington Heights, IL) and DMSO from Aldrich (St. Louis, MO). Commercial and consumer products were obtained from local department stores and laboratory product suppliers. The sources of the materials examined in detail are as follows: newspaper (Davis Enterprise, Davis, CA), business card (Kinkos, Davis, CA), blue paper towel (Georgia-Pacific professional), yellow legal writing pad (Universal Office Products, Waterford, NY), FisherBrand rubber cell scraper (Walter Stern, Inc., Port Washington, NY), black 0-ring (Danco Co., Irving, TX), FisherBrand black rubber stopper (Plasticoid, Elkton, MD), red rubber band (OfficeMax, Davis, CA). The indicated commercial and consumer products were finely diced with scissors and extracted for 24 hr in Teflon-capped glass tubes containing dimethylsulfoxide (DMSO), ethanol (ETOH, 95%), or Milli-Q water using 1.5 ml of solvent for each gram of sample with the exception of the paper products which were extracted with 9 volumes of solvent per gram of sample due to absorption of the solvent by the paper. After centrifugation, supernatants (extracts) were transferred into Teflon-capped glass vials and stored in the dark until use.

### Preparation of cytosol and DNA and ligand binding analysis

Male Hartley guinea pig (500 g, Charles River Laboratories) hepatic cytosol was prepared and used in gel retardation analysis experiments to measure DNA binding of *in vitro* transformed AhR complexes and in hydroxyapatite assays to measure competitive [^3^H]TCDD ligand binding analysis as described in detail [14]. For gel retardation analysis, cytosol (8 mg protein/ml) was incubated with DMSO (20 µl/ml, final concentration), 20 nM TCDD or the indicated extract (20 µl/ml) for 2 hr at 20°C and ligand-activated protein-DNA complexes (AhR∶ARNT (AhR nuclear translocator)∶DRE (dioxin responsive element)) were resolved in non-denaturing PAGE gels and quantitated using a Molecular Dynamics Phosphorimager [14]. The amount of ligand-activated AhR∶DRE complex formation was expressed relative to that produced by TCDD. For ligand binding, cytosol (2 mg protein/ml) was incubated with 2 nM [^3^H]TCDD in the absence or presence of 200 nM TCDF, DMSO (10 µl/ml, final concentration) or the indicated extract (10 µl/ml) for 2 hours in a room temperature water bath. [^3^H]TCDD binding in aliquots of the incubation (200 µL) was determined by HAP binding as previously described [14]. The total amount of [^3^H]TCDD specific binding was obtained by subtracting the non-specific binding ([^3^H]TCDD and TCDF) from the total binding ([^3^H]TCDD). The ability of a chemical(s) in a sample extract to bind to the AhR was indicated by its ability to competitively reduce [^3^H]TCDD specific binding.

### Cell cultures, chemical treatment, luciferase activity and cytochrome P4501A1 (CYP1A1) mRNA

For AhR-dependent chemically activated luciferase expression (CALUX) analysis, recombinant guinea pig intestinal adenocarcinoma (G16L1.1c8), mouse hepatoma (H1L1.1c2), rat hepatoma (H4L1.1c4) and human hepatoma (HG2l1.1c3) cells, containing the stably integrated DRE-driven firefly luciferase reporter plasmid pGudLuc1.1, were grown, maintained, treated and luciferase activity measured as previously described in detail [13], [15]. Luciferase activity in each well was expressed relative to that induced by 1 nM TCDD. Analysis of the time-dependence of the ability of each extract to stimulate AhR-dependent gene expression was carried out using a combination of recombinant mouse hepatoma (Hepa1c1c7) CALUX cell lines (H1L1.1c2 and H1L6.1c2). These two cell lines are identical except that each contains a slightly different stably integrated AhR-/DRE-driven firefly luciferase plasmid (pGudLuc1.1 or pGudLuc6.1, respectively). The minor differences in these two reporter plasmids results in cell lines whose time course of luciferase induction varies as a result of differences in intracellular localization and stability of the Promega luciferase gene product [16]. While the H1L1.1c2 cells induce maximally by 4–6 hours after agonist treatment, H1L6.1c2 cells induce more slowly with little activity at 4–6 hours and with maximal induction by 24 hours after agonist treatment [15], [17]. The H1L1.1c2 cells are optimal for identifying AhR activators that are metabolically unstable and thus exhibit maximal induction within a few hours after addition to cells, while H1L6.1c2 cells are optimal for identifying AhR activators that are more metabolically stable and induce gene expression over a longer period of time [17]–[19]. For time-dependent analysis, cells grown in white clear-bottomed 96-well microplates were incubated with carrier solvent DMSO (10 µl/ml), TCDD (1 nM), or the indicated concentration of sample extract for 4 h (in H1L1.1c2 cells) or 24 h (in H1L6.1c2 cells) at 37°C and luciferase activity measured in a microplate luminometer as previously described [13]. For analysis of AhR-responsive endogenous CYP1A1 gene expression, mouse hepatoma (Hepa1c1c7) cells were treated with the indicated chemical/extract and mRNA for CYP1A1 and Rig-S15 (internal control for normalization using primers 5′-TTCCGCAAGTTCACCTACC and 5′-CGGGCCGGCCATGCTTTACG) amplified by RT-PCR as previously described in detail [14].

Analysis of the ability of extracts to stimulate estrogen receptor (ER)-dependent gene expression, we utilize a recombinant human ovarian (BG1Luc4E2) cell line that contains a stably transfected ER-responsive firefly luciferase reporter gene [20]. Cells were grown and maintain as described [20] and were maintained in estrogen-stripped media for 5 days before they were plated into white, clear-bottomed 96-well tissue culture dishes at 75,000 cells/well and allowed to attach for 24 hr. Cells were then incubated with carrier solvent (ethanol; 1% final solvent concentration), 17β-estradiol (1 nM), the indicated sample extract for 24 hr at 37°C. Luciferase activity was determined as described above and activity expressed relative to that produced by E2.

### Exposure to human skin and quantitative real time PCR

On the day of excision, samples of human foreskin were cut in half and suspended in 4 ml of DMEM∶F12 (2∶1) culture medium. The samples were obtained as discarded (routinely collected earlier that day in the circumcision clinic), and no information about the subject was available. Collection without informed consent was approved by the University of California, Davis Institutional Review Board under exempt category #4. For each tissue sample, one half was treated with DMSO (20 µl/ml) and the other with DMSO (20 µl/ml) containing TCDD (final concentration 10 nM) or the indicated extract (20 ml/ml). After overnight incubation at 37°C, samples were rinsed quickly in phosphate buffered saline, frozen in liquid nitrogen, homogenized to a powder in a mortar and pestle and extracted with Trizol. Total RNA was transcribed with a High Capacity cDNA Archive kit (Applied Biosystems, Carlsbad, CA), and cDNAs were quantitated using Applied Biosystems Taqman gene expression assays for CYP1A1 (Hs00153120_m1), b-actin (Hs99999903_m1) and 18S RNA (Hs99999901_s1) with an ABI 7700 real time PCR Sequence Detection System as previously described [21]. Relative mRNA levels were calculated using the Ct method and CYP1A1 expression was normalized to the geometric mean of b-actin and 18S RNA values [22].

### Zebrafish embryo ethoxyresorufin O-deethylase (EROD) assay, CYP1A antisense morpholino injection and mophological analysis

Newly fertilized AB* zebrafish embryos were placed in egg-water (60 µg/L Instant Ocean Salts, Aquarium Systems, Mentor, OH) containing DMSO (0.02% (v/v)), 1∶5,000 newspaper DMSO extract or 1∶5,000 or 1∶20,000 rubber stopper DMSO extract for 96 hours and maintained at 28°C on a 14 hr light, 10 hr dark cycle. Some embryos were injected 2 h postfertilization with 3 nl of a solution containing zebrafish CYP1A antisense morpholino DNA (zfcyp1a-MO; 5′-TGGATACTTTCCAGTTCTCAGCTCT-3′) that was 0.1 mM in Danieau's solution. All hatched larvae were anesthetized with MS222, collected and mounted in 3% methyl cellulose and visualized with brightfield microscopy for developmental defects and with fluorescent microscopy for CYP1A-mediated EROD activity [23]. These experiments were all with embryos and as such were not under any IACUC protocol as they were not considered vertebrate animals, only embryos, and as such there were no IACUC guidelines or protocol. The embryos were obtained from adult fish under Duke University's IACUC program. Adult zebrafish care and reproductive techniques were non-invasive and have been reviewed and approved by Duke University Institutional Animal Care and Use Committee under protocol #A279-08-10.

## Results and Discussion

While the best-studied and highest affinity ligands for the AhR are a variety of HAHs and polycyclic aromatic hydrocarbons (PAHs), the AhR and AhR signal transduction pathway can be activated by a structurally diverse range of chemicals [2], [3], [5], [6], [24]. The documented promiscuity of AhR ligands suggests that there actually exists a much larger spectrum of structurally diverse AhR ligands than has been currently identified. In fact, recent studies examining the ability of crude polar and nonpolar solvent extracts of a variety of environmental, biological, food and commercial and consumer products are consistent with this hypothesis [10]–[13]. To extend our previous studies demonstrating the presence of AhR agonists in crude extracts of newspapers and tires, we carried out preliminary screening analysis of DMSO extracts of a wide variety of commercial or consumer products using gel retardation analysis. These experiments (Figure S1) revealed that the majority of these extracts contained chemicals that could stimulate transformation and DNA binding of guinea pig hepatic cytosolic AhR. In order to more fully characterize the AhR activity of chemicals contained in extracts of commercial and consumer products, we focused our analysis on extracts of a smaller subset of these materials, namely paper products (newspaper, business cards, blue paper towel, yellow writing paper) and rubber products (cell scraper, black o-ring, black stopper and red rubber band).

**Figure 1:**
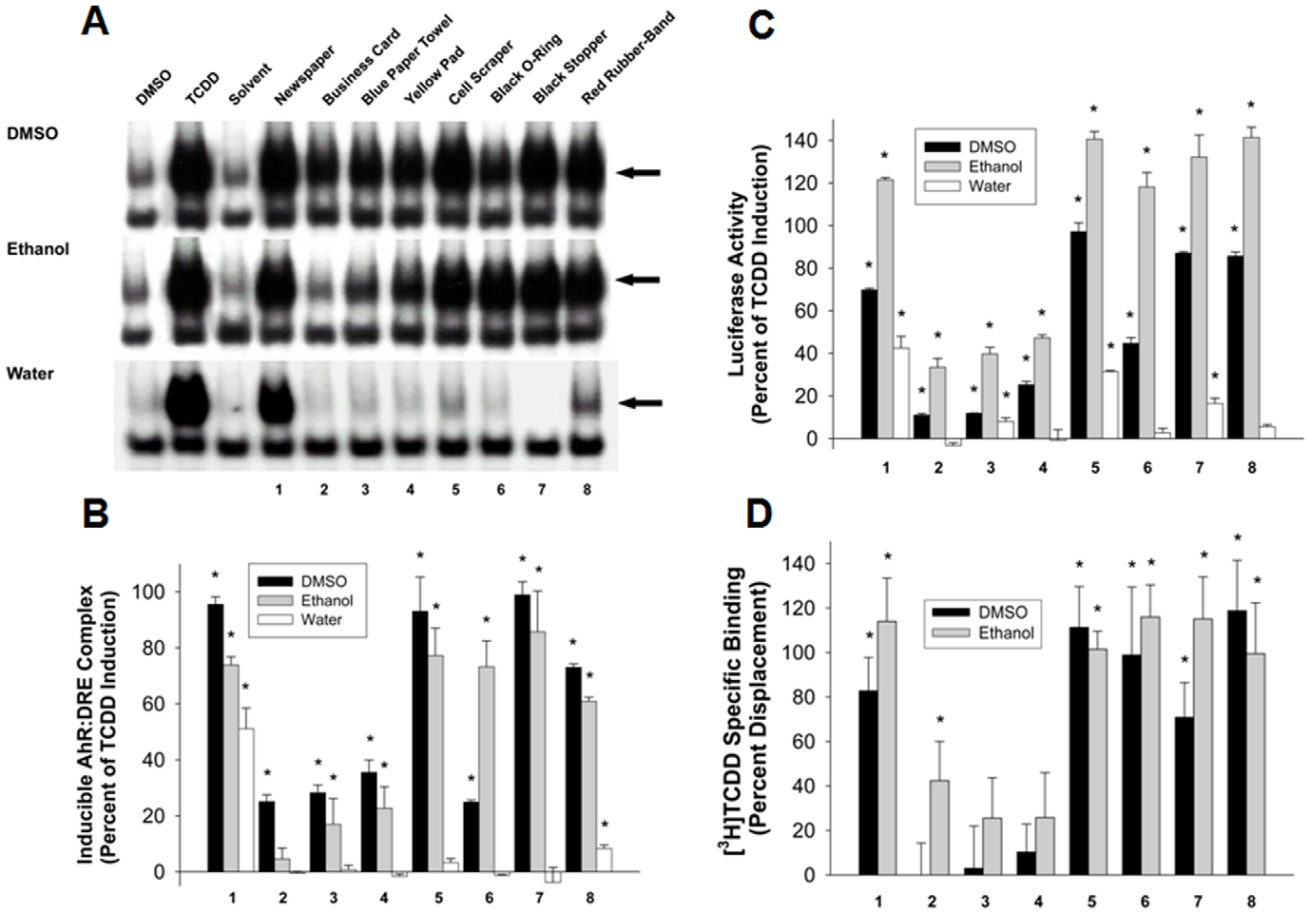
Activation of the AhR and AhR-dependent signal transduction pathway by DMSO, ETOH and water extracts of commercial and consumer products. The products used in these studies were (1) newspaper (black print section only); (2) business card; (3) blue paper towel; (4) yellow pad; (5) cell scraper; (6) black rubber O-ring; (7) black rubber stopper; (8) red rubber-band. (A) Stimulation of AhR transformation and DNA binding by extracts of the indicated commercial and consumer products in vitro. The arrow indicates the position of the ligand-activated protein-DNA (AhR∶ARNT∶DRE) complex in the gel retardation assay and results are representative of three individual experiments. (B) The amount of ligand-activated protein-DNA complex formation from gel retardation experiments from part A was determined by phosphorimager analysis. Values are expressed as the percentage of maximal TCDD induction and represent the mean ± SD of all DNA binding data compiled from duplicate gels from three individual experiments. Values significantly different from solvent alone (p≤0.05 as determined by the students T-test) are indicated by an asterisk. (C) Induction of luciferase activity by individual extract in recombinant guinea pig (G16L1.1c8) cells. Cells were incubated with the indicated extract (10 µl/ml) for 4 h and luciferase activity determined as described in Materials and Methods. Values are expressed as a percentage of the maximal induction by TCDD and represent the mean ± SD of triplicate determinations and those values significantly different from solvent alone (p≤0.05 as determined by the students T-test) are indicated by an asterisk. The results shown are representative of three individual experiments. (D) Competitive binding between [^3^H]TCDD and the indicated DMSO or ethanol extract for the guinea pig hepatic cytosolic AhR. Guinea pig cytosol was incubated with 2 nM [^3^H]TCDD in the absence or presence of the indicated extract for 2 h at 20°C, and specific binding of [^3^H]TCDD was determined by hydroxyapatite binding. Values represent the mean ± SD of triplicate determinations and are representative of three individual experiments and those values significantly different from solvent alone (p≤0.05 as determined by the students T-test) are indicated by an asterisk.

To characterize the AhR agonists contained in these materials, we extracted paper and rubber products with various solvents (DMSO, ETOH, water) and tested the ability of the extracts to stimulate AhR transformation and DNA binding in vitro using gel retardation analysis (Figure 1A, B). All of the DMSO extracts stimulated high amounts AhR∶DNA complex formation, and most ethanol extracts were also active (particularly those of newspaper and all rubber products tested), stimulating AhR DNA binding to 60–80% of that of a maximally activating concentration of TCDD. It should be noted that similar materials from other suppliers/manufacturers have also been examined in this assay with comparable results (data not shown), indicating that the AhR agonist activity of extracts of these materials is not specific to a single supplier. We previously detected AhR agonists in ethanol and DMSO extracts of newspaper ink and newspapers from throughout the world [25], although attempts to identify the responsible chemicals have not yet been successful (data not shown). While AhR agonists are typically very hydrophobic, the high degree of AhR transformation and DNA binding observed after incubation of hepatic cytosol with the water extract of printed newspaper and to a lesser extent by that of the red rubber band (Figure 1A, B) indicate the existence of novel water soluble AhR agonists. Taken together, the presence of AhR agonist activity in DMSO, ethanol and water extracts suggests the existence of AhR agonists with a variety of physicochemical characteristics in the tested commercial and consumer product extracts.

Since the ability of a compound or extract to induce AhR transformation in vitro does not always correlate with its ability to activate the AhR signal transduction pathway [12], we next examined the ability of these extracts to induce gene expression in a guinea pig adenocarcinoma cell line containing a stably transfected AhR-responsive luciferase reporter gene [15] (Figure 1C). The ability of the extracts to induce AhR-dependent gene expression in these cells compared well with their ability to stimulate guinea pig AhR transformation and DNA binding in vitro (compare Figures 1B and 1C). Interestingly, while the ethanol extracts were either equipotent to or less potent than the DMSO extracts in the DNA binding assays (Figure 1B), luciferase reporter gene induction by ethanol extracts was greater than that of DMSO extracts of the same material (Figure 1C) and suggests that ethanol extracts a different subset of AhR agonists from the materials that have a greater affinity for the AhR and/or produces a more efficacious induction response. Interestingly, the magnitude of reporter gene induction by the ethanol extracts of newspaper (sample 1) and rubber products (samples 5–8) was also considerably greater than that obtained with a maximally inducing concentration of TCDD. “Superinduction” of AhR-dependent gene expression by selected chemicals and crude extracts of environmental samples has been previously reported by several laboratories. While the exact molecular mechanisms responsible for the effect have not been elucidated, it has been attributed previously to cross-talk between the AhR and components of cell signaling (i.e. protein kinase C) and protein degradation pathways [26]–[29]. Similar to the DNA binding assay results, these analyses also revealed that water extracts of newspaper and select rubber products (cell scraper and black stopper, samples 5 and 8, respectively) contain polar AhR agonists that can activate AhR-dependent gene expression in intact cells.

Examination of the ability of DMSO and ETOH extracts to compete directly with [^3^H]TCDD for binding to the guinea pig hepatic cytosolic AhR revealed that all of the extracts, except for the DMSO extracts of paper products (i.e., yellow pad, blue paper towel and business card), could competitively bind to the AhR and are thus full agonists (Figure 1D). Little or no competitive binding was observed with the water extracts (data not shown). The ability of the DMSO and water extracts of paper products to directly stimulate AhR transformation and DNA binding as well as AhR-dependent luciferase induction, but to show little or no competitive ligand binding activity, suggests that they have relatively low affinity for the AhR and thus are not able to compete effectively with the high affinity ligand [^3^H]TCDD. We previously observed this phenomenon with other weak AhR agonists [6], [8], [11].

**Figure 2:**
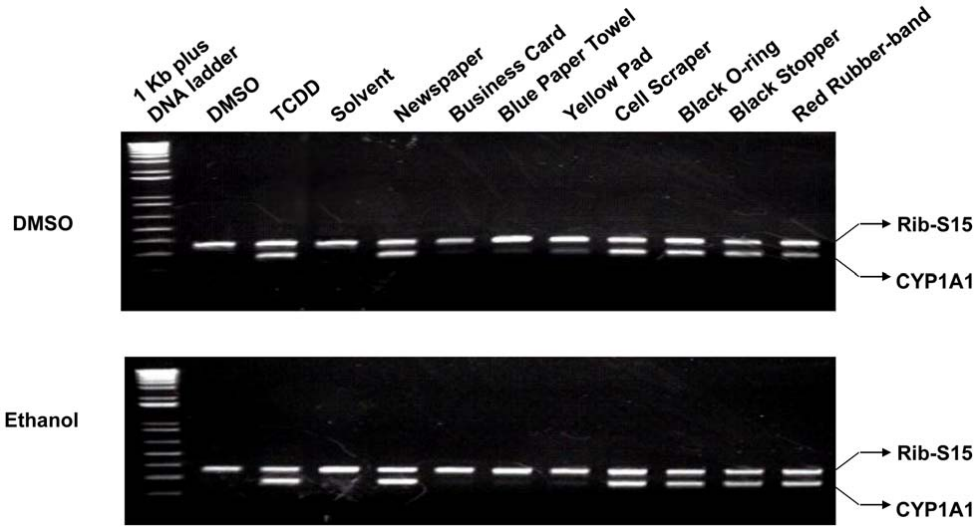
Effect of extracts of commercial and consumer products on CYP1A1 mRNA levels in mouse hepatoma cells. Hepa1c1c7 cells were incubated with DMSO (10 µl/ml), TCDD (1 nM in DMSO) or the indicated extract (20 µl/ml) for 3.5 hr at 37°C, mRNA was extracted, subjected to RT-PCR and the resulting products were visualized by agarose gel electrophoresis. PCR amplification of CYP1A1 and ribosomal S15 (the loading control) from these samples was carried out as described in Materials and Methods and the results shown are representative of two separate experiments.

**Figure 3:**
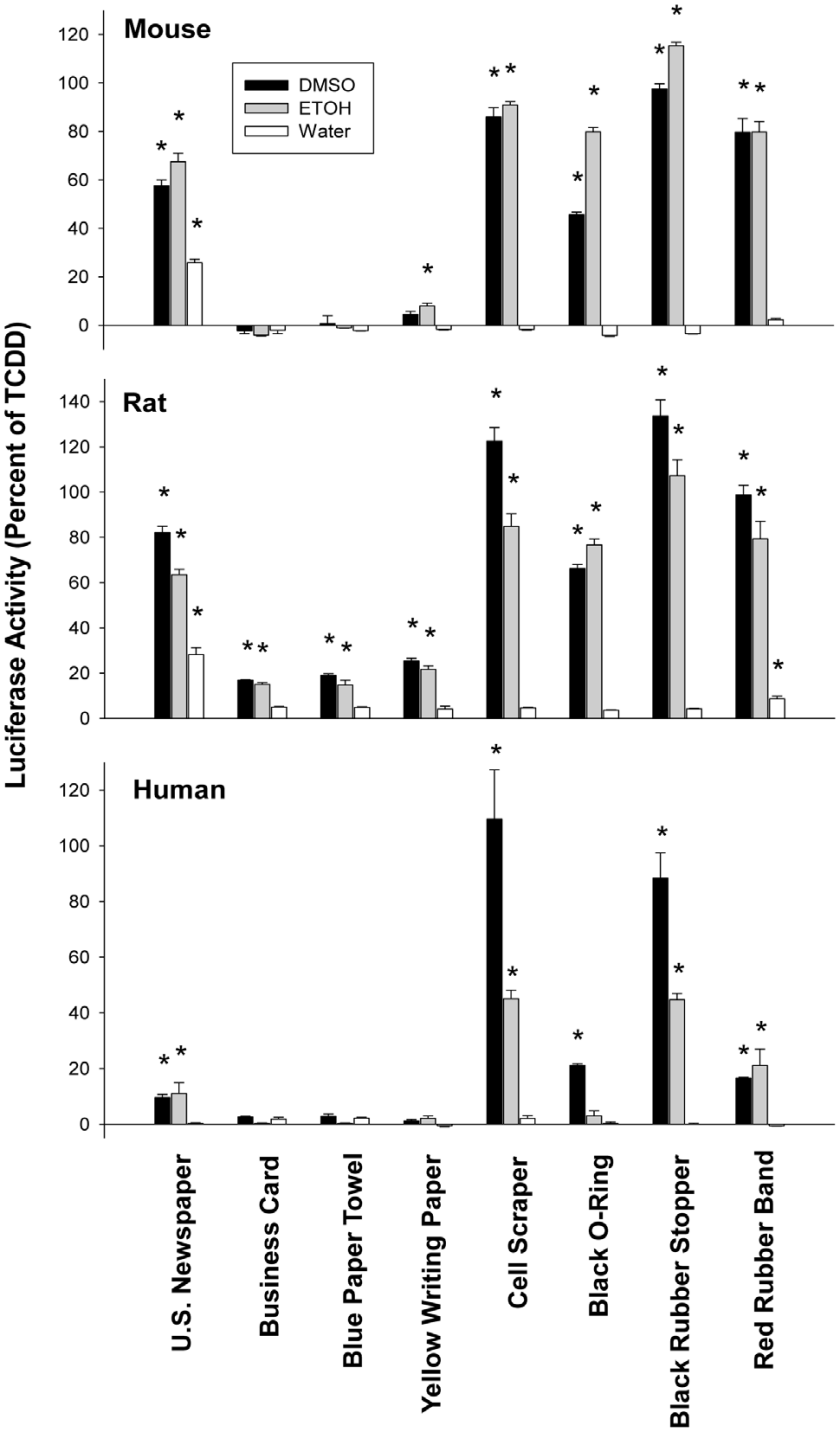
Induction of AhR-dependent luciferase reporter gene activity in stably transfected mouse, rat and human hepatoma cells by extracts of commercial and consumer products. Recombinant mouse (H1L1.1c2), rat (H4L1.1c4) and human (HG21.1c3) hepatoma cell lines were incubated with the indicated extract (10 µl/ml) for 4 hr and luciferase activity determined as described in Material and methods. Values are expressed as a percentage of the maximal luciferase induction by TCDD and represent the mean ± SD of triplicate determinations. The results shown are representative of duplicate experiments and those values significantly greater than that of solvent alone (p≤0.05 as determined by the students T-test) are indicated by an asterisk.

The induction response was also characterized with respect to incubation time, effect on endogenous CYP1A1 and effectiveness in several species. First, mouse hepatoma CALUX cell luciferase induction response was compared at 4 hours versus 24 hours of incubation (Figure S2). The lower luciferase activity evident at the later time point is consistent with the AhR agonists present in the extracts as being metabolically labile. Additionally, since the AhR agonist activity/potency of our DMSO extracts was not reduced if the vials containing the extracts were left open for a day, the reduction in gene induction over time was unlikely to be due to evaporative loss of the AhR agonists during incubation (data not shown). In contrast, we observed little or no loss of luciferase induction potency of these extracts when they were stored at room temperature in the dark for up to one year (data not shown), indicating that these agonists are chemically stable. Second, the ability of DMSO and ETOH extracts to stimulate expression of an endogenous AhR-responsive gene (CYP1A1) was confirmed by demonstrating an increase in mRNA levels in mouse hepatoma (hepa1c1c7) cells using RT-PCR. Incubation of cells with DMSO or ETOH extracts (1∶100 (v/v) dilution) of rubber products, newspaper, or yellow-pad paper for 3.5 hr resulted in increased CYP1A1 mRNA levels (Figure 2), and these results were directly comparable with the ability of each extract to induce AhR-dependent luciferase activity (Figure 1C). Finally, although these extracts could stimulate AhR-dependent luciferase gene induction in recombinant mouse, rat and human hepatoma cells (Figure 3), significant species differences in response to different extracts were also observed. Species-specific differences in ligand-selectivity of the AhR have been previously observed [3], [30]–[32] and likely result from differences in the specificity and affinity of binding of ligands as well as from variations in the metabolic activity of each cell line.

**Figure 4:**
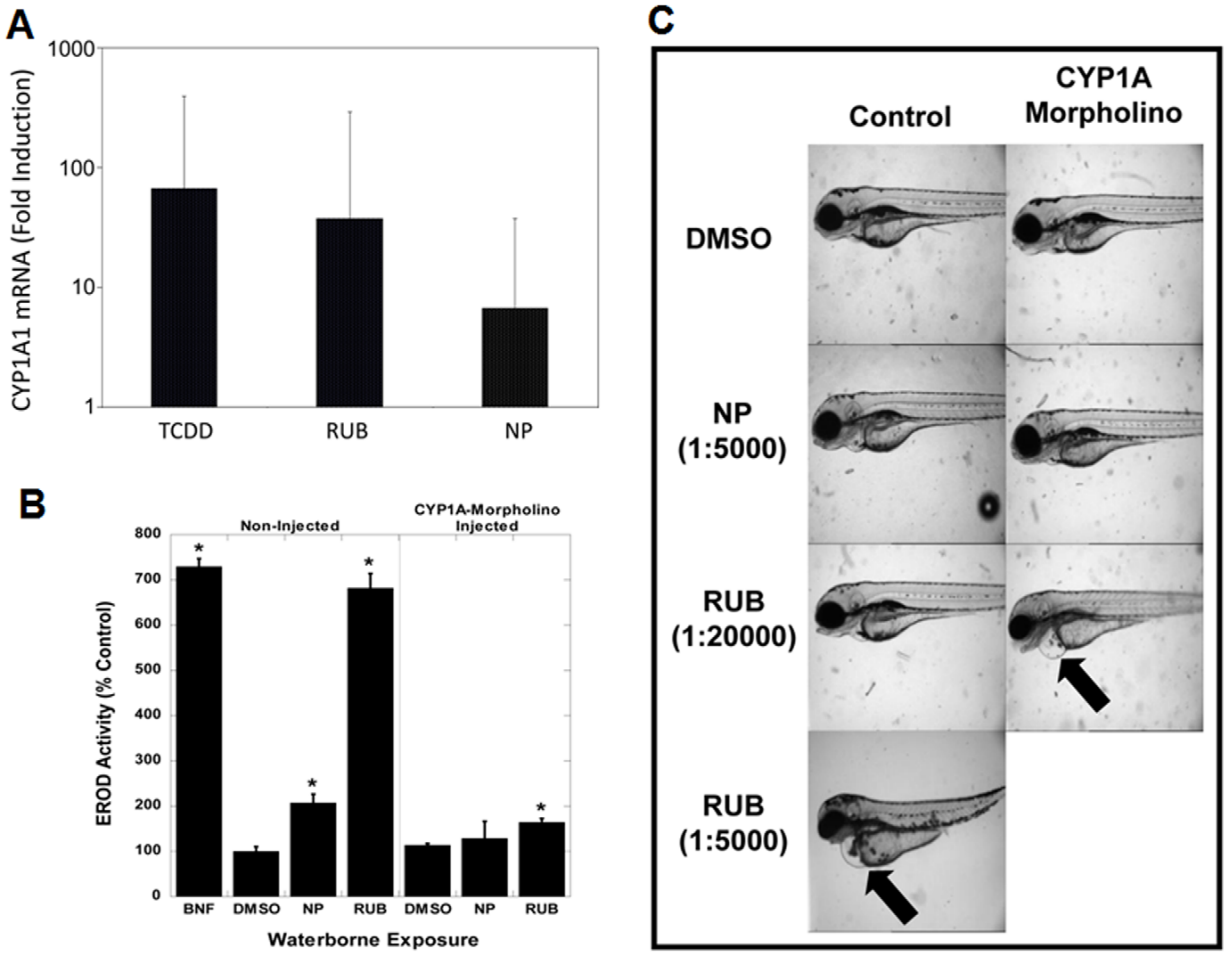
Effect of extracts of newspaper and rubber on human skin CYP1A1 mRNA, embryonic zebrafish CYP1A-dependent EROD activity and zebrafish development. (A) Human skin was incubated with the indicated extract (newsprint (NP) or rubber stopper (RUB) at 1% final concentration) overnight at 37°C, mRNA was isolated and transcribed into cDNA and quantitated by real time PCR. Values are expressed as the mean ± SD of 4 (TCDD) or 6 (extract) individual skin samples. All values were significantly different from those of DMSO controls (set to 1) at p<0.05 as determined by one-way ANOVA using Stata/SE9.2 software for Windows with Bonferroni corrections. (B) Newly fertilized zebrafish embryos were exposed for 96 h to DMSO (0.02% v/v), newspaper (NP) extract (1∶5,000 dilution), or rubber (RUB) stopper (1∶5,000 or 1∶20,000 dilution) added to the water and some also injected with 2 pumps of 1× Danio embryo water or embryo water containing 0.15 mM CYP1A-morpholino; additional embryos were exposed to the AhR agonist beta-naphthoflavone (BNF, 1 µg/L) as the positive control for the same period. Hatched larvae were collected and analyzed for EROD activity. EROD values are expressed as the mean ± SE of 5 embryos, where the asterisk indicates those values significantly different from the DMSO control at p<0.05 as determined by Student's t-test. (C) The hatched larvae treated with extracts as in Figure 5B were examined for deformities by brightfield microscopy.

Taken together, our results indicate that chemicals present in these extracts can bind to the AhR, stimulate AhR DNA binding and consequently induce gene expression in vitro and in continuous cell lines in culture. The physiological, biochemical and/or toxicological significance of these results would be strengthened by demonstrating their ability to activate the AhR in tissues and animals in vivo. Accordingly, we examined the ability of two of the most potent samples (DMSO extracts of newspaper and rubber stopper) to stimulate AhR-dependent CYP1A1 expression in normal human skin and in zebrafish in vivo. When fresh human foreskin was exposed in organ culture to TCDD or to DMSO extracts of newspaper or rubber stopper, real-time PCR revealed the ability of these extracts to increase CYP1A1 mRNA levels (Figure 4A), with the relative increase in mRNA comparable to their luciferase induction potency (Figure 1). These results demonstrate that the more potent DMSO extracts can increase expression of the endogenous AhR-responsive CYP1A1 gene in normal human skin. To examine induction of CYP1A1-dependent EROD activity by these extracts in vivo, zebrafish were exposed to DMSO extracts of newspaper and rubber stopper in egg water and EROD activity determined (Figure 4B). We previously used zebrafish embryos to demonstrate the ability of PAHs and other metabolically labile AhR agonists both to stimulate this activity and to produce AhR-dependent toxicity in vivo [23], [25], [33]. Exposure of zebrafish to a 1∶5,000-fold dilution of the DMSO newspaper extract in egg-water resulted in approximately a two-fold increase in EROD activity compared to the DMSO control, while that of a 1∶20,000-fold dilution of the rubber stopper DMSO extract resulted in a 6.8-fold increase in EROD activity (Figure 4B). β-Naphthoflavone, a classical AhR agonist (at 1 µg/L in egg water), induced EROD activity 7-fold.

At the low extract concentrations above, no adverse morphological effects in the zebrafish were observed. Increasing the concentration of rubber extract to 1∶5,000 in egg water resulted in substantial malformations in all fishes, including pericardial edema (see arrow in Figure 4C), yolksac edema, necrosis, cranial edema, and severe craniofacial malformations especially of the lower jaw. The increase in EROD activity in zebrafish exposed to newspaper or rubber stopper extract was the result of an increase in CYP1A, as EROD induction was blocked when zebrafish were injected with an antisense CYP1A morpholino (Figure 4B) previously shown to inhibit increases in CYP1A mRNA, protein and EROD activity [23]. Interestingly, while no adverse developmental effects were observed in the zebrafish exposed to a 1∶20,000-fold dilution of rubber stopper extract in their water, when these fish were also injected with the CYP1A antisense morpholino, dramatic adverse developmental effects were observed, comparable to fish exposed to a 1∶5,000-fold dilution of the rubber extract (Figure 4C). These effects likely resulted from a decrease in CYP1A-dependent degradation of the chemical(s) in the extract responsible for the adverse effects. Thus, chemicals present in these extracts can induce AhR-dependent gene expression and toxicity in zebrafish in vivo.

**Figure 5:**
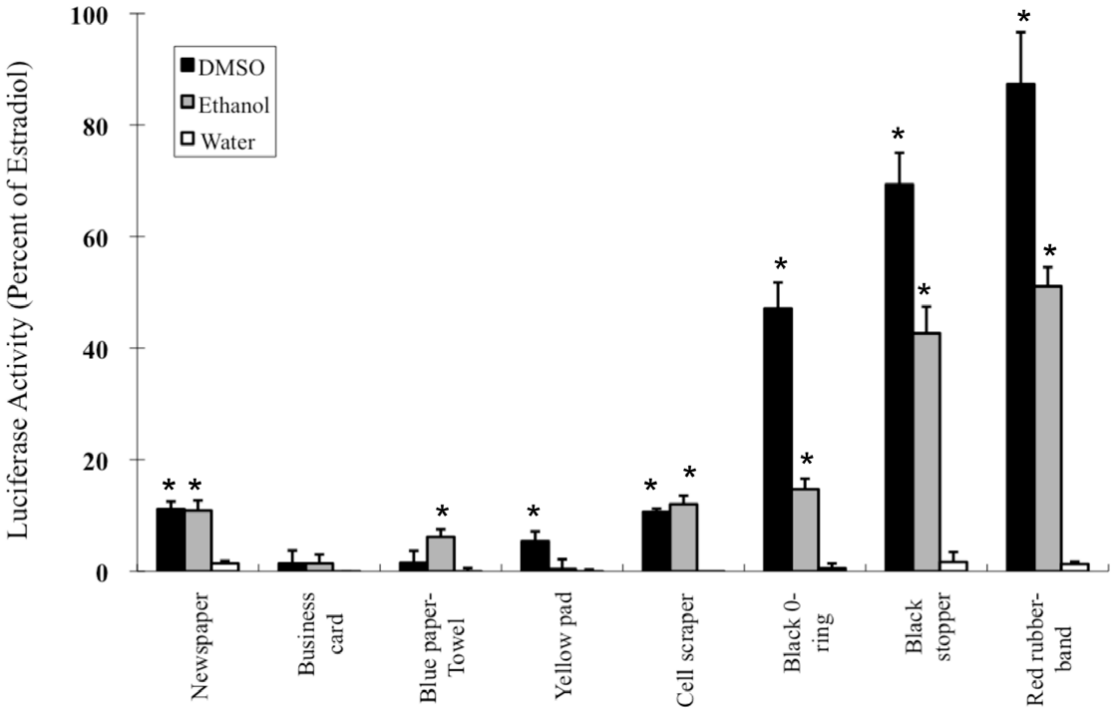
Estrogen reporter stimulation by rubber extracts. Human ovarian BG1Luc4E2 cells, containing a stably transfected estrogen receptor responsive reporter, were treated with the indicated extracts as previously described (Rogers and Denison, 2000). Induction of luciferase is shown in comparison with maximal induction by 17β-estradiol (1 nM in ethanol) after incubation for 24 h. Values represent the mean ± SD of at least triplicate incubations and the results shown are representative of three individual experiments. Values significantly different from solvent alone (p≤0.05 as determined by the students T-test) are indicated by an asterisk.

Exposure to AhR agonists can result in a variety of species- and tissue-specific toxic and biological effects [2], [4], [5], [23], [33]. While our results indicate that the AhR active chemicals extracted from these materials are evidently biotransformed, they are able to produce TCDD-like toxic and biological effects observed in animals exposed to metabolically stable AhR agonists [2], [4], [5], [23], and chronic exposure could produce other AhR-dependent adverse effects. For example, activation of the AhR can produce endocrine disrupting effects on a variety of hormone receptor systems, from alterations in hormone synthesis and degradation, to reductions in hormone (particularly estrogen) responsiveness [1], [20], [34]. Since some of these commercial and consumer product extracts contain substantial AhR agonist activity, it is possible that they can also contain chemicals that can produce endocrine disrupting effects in exposed animals. Since these extracts are likely complex mixtures of chemicals, and numerous structurally diverse xenobiotics have been shown to bind to and activate steroid hormone receptor signaling pathways [35]–[38], it is likely that these extracts also contain steroid hormone receptor agonists. In fact, DMSO and ETOH extracts of the rubber products produce a substantial estrogenic induction response in a recombinant human ovarian carcinoma (BG1) cell line (BG1Luc4E2) containing a stably transfected estrogen receptor responsive luciferase reporter gene [38], demonstrating that they contain estrogen receptor agonists (Figure 5). While endocrine disrupting chemicals (EDCs) have been identified in extracts of environmental and food matrices, personal care products, sunscreens and a limited number of commercial and consumer products [39]–[42], most studies have focused on identification of known EDCs, rather than assessing the overall EDC activity of a sample extract and then identifying the responsible chemicals. Using hormone receptor based screening approaches, like that described here for the AhR, extracts of a very limited number of paper, rubber and plastic materials have been previously shown to contain estrogenic, antiestrogenic, androgenic, and/or antiprogesteronic activity [43]–[45]. Thus, in addition to AhR agonists, commercial and consumer products also contain extractable estrogenic EDCs. The effect of these extracts on other nuclear receptor signaling pathways remains to be determined.

While the identities of the AhR- and ER-active chemicals described here and their toxicological impacts remain to be determined, their ease of extractability from everyday products suggests they may interfere with bioanalytical methods for quantitation of dioxin- and estrogen-like chemicals. In vitro and cell-based (CALUX-like) AhR- and ER-dependent bioassays have gained widespread acceptance as rapid and inexpensive methods for relative quantitation of dioxin- and estrogen-like chemicals in environmental, biological, food and commercial products [38], [46]–[49]. Thus AhR/ER agonists, present in materials with which samples come into contact, could be transferred to a test sample, thus artifactually increasing its overall response. AhR/ER agonists could even be directly extracted from a non-glass storage container or from its cap or liner (not Teflon) by a hydrophobic sample material itself. For example, use of vacutainers with rubber stoppers for blood/serum samples might result in artifactual elevation in AhR/ER agonist activity. Bioanalytical methods commonly detect greater levels of dioxin- and estrogen-like activity than can be confirmed by standard analytical quantitation of known AhR and ER constituents. Thus, the background of extractable AhR/ER agonists in sample collection and processing materials must be determined using method blanks.

In conclusion, the studies described here demonstrate that extracts of commercial and consumer products contain agonists of the AhR and estrogen receptor signal transduction pathways. A major question that remains is the identity of the chemicals present in these extracts responsible for agonist activity. While the AhR agonist activity of some of these extracts could result, at least in part, from known AhR-active chemicals that are specifically added to these materials during their manufacture (i.e. artificial dyes and colorants [50], [51], plasticizers [52], [53] and other chemicals [13]), the documented promiscuity of ligand binding by these receptors, coupled with the demonstration of agonist activity in DMSO, ethanol and water extracts of these materials, suggests that the extracts contain numerous agonists with a variety of physicochemical characteristics. Similarly, while our previous bioassay-directed chemical fractionation allowed us to identify a variety of PAHs and novel benzothiazole agonists from automobile tires, they also indicated the presence of additional physicochemically diverse agonists [13]. The identification of AhR and estrogen receptor agonists in extracts of commercial and consumer products is a primary direction for future studies. The results will not only expand our knowledge of agonists and EDCs present in common commercial and consumer products, but also allow critical evaluation of their biochemical and toxicological significance.

## Supporting Information

Figure S1 **Stimulation of in vitro AhR transformation and DNA binding of guinea pig hepatic cytosolic AhR by DMSO extracts of commercial and consumer products.** The extracts were prepared as described in Material and Methods. The arrow indicates the position of the ligand-activated protein-DNA (AhR∶ARNT∶DRE) complex in the gel retardation assay and the results shown are representative of three individual experiments.
(TIF)

Figure S2 **Time dependence of AhR gene induction response.** Recombinant mouse hepatoma cells were incubated with equal amounts of the indicated extracts for 4 h (H1L1.1c2 cells - left panel) or 24 h (H1L6.1c2 cells - right panel) and luciferase activity determined as described in Materials and Methods. In each case, the values were normalized to the response obtained with TCDD and expressed the mean ± SD of at least triplicate determinations. Values significantly different from solvent alone (p≤0.05 as determined by the students T-test) are indicated by an asterisk.
(TIF)
