# Supplementary figures and images for: Common Commercial and Consumer Products Contain Activators of the Aryl Hydrocarbon (Dioxin) Receptor
Source: PLoS One. 2013 Feb 18;8(2):e56860. doi: 10.1371/journal.pone.0056860 (PMC3575475; doi:10.1371/journal.pone.0056860)

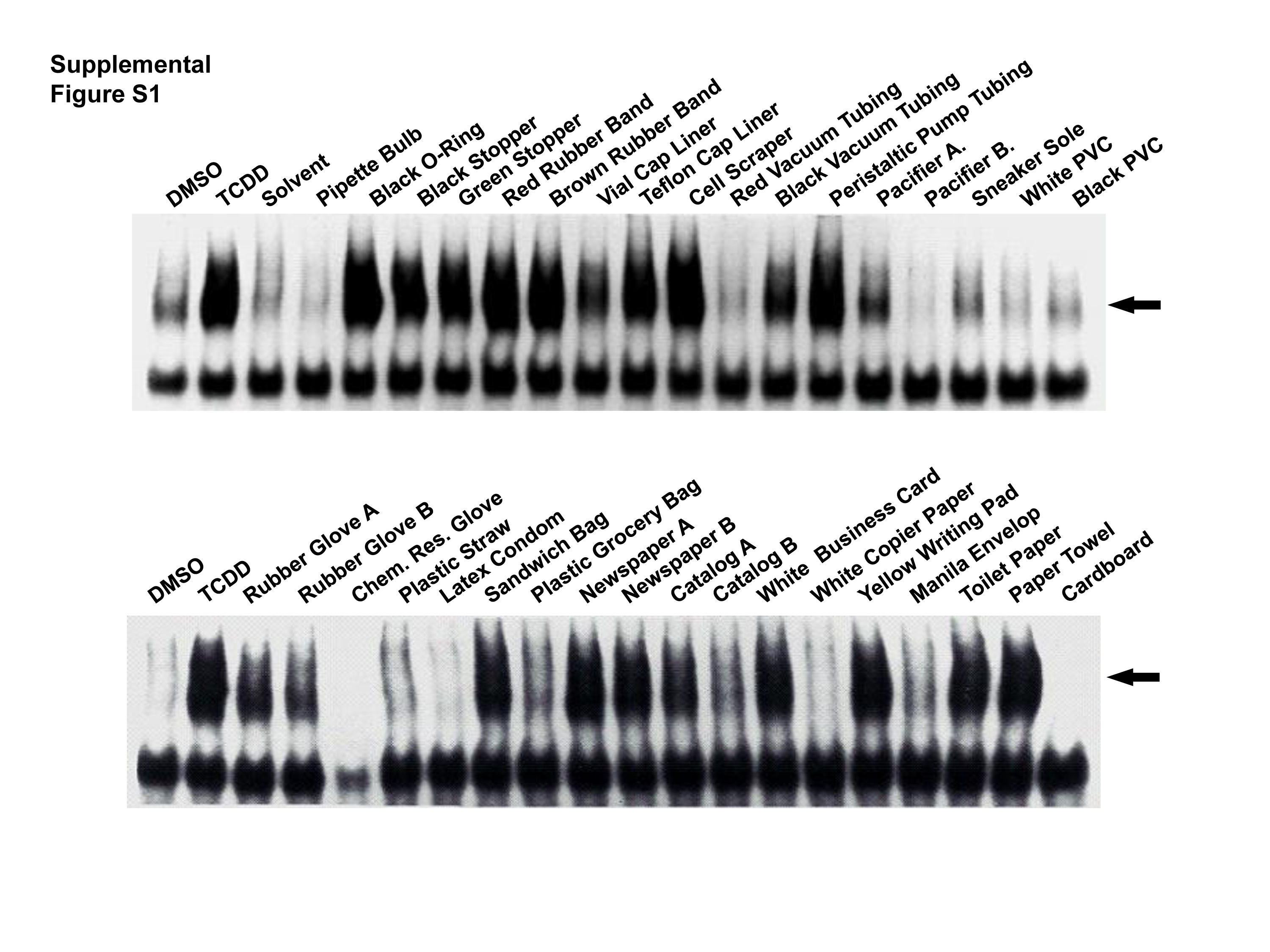

Supplement: Figure S1 — Stimulation of in vitro AhR transformation and DNA binding of guinea pig hepatic cytosolic AhR by DMSO extracts of commercial and consumer products. The extracts were prepared as described in Material and Methods. The arrow indicates the position of the ligand-activated protein-DNA (AhR∶ARNT∶DRE) complex in the gel retardation assay and the results shown are representative of three individual experiments. (TIF) [file pone.0056860.s001.tif]

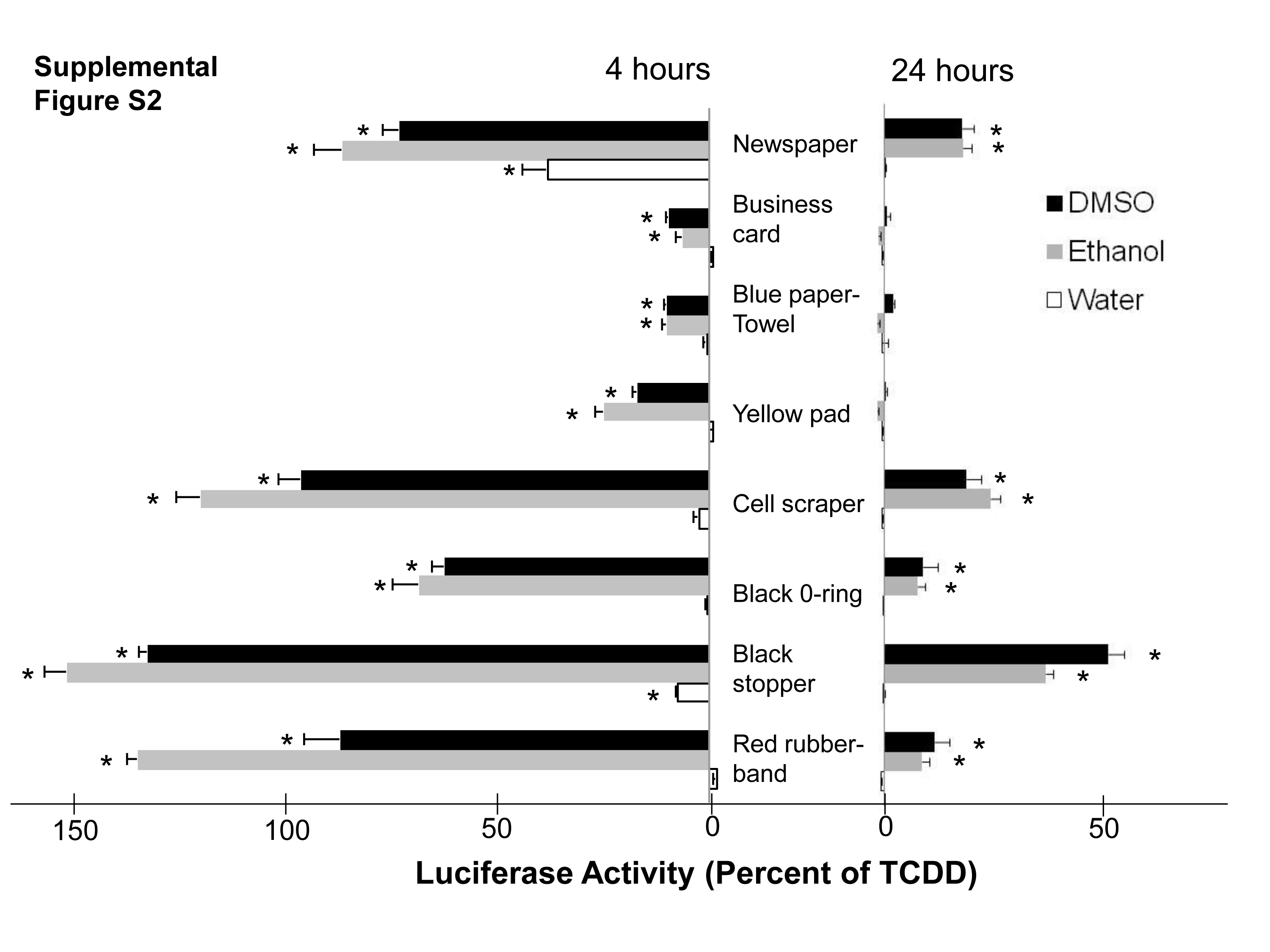

Supplement: Figure S2 — Time dependence of AhR gene induction response. Recombinant mouse hepatoma cells were incubated with equal amounts of the indicated extracts for 4 h (H1L1.1c2 cells - left panel) or 24 h (H1L6.1c2 cells - right panel) and luciferase activity determined as described in Materials and Methods. In each case, the values were normalized to the response obtained with TCDD and expressed the mean ± SD of at least triplicate determinations. Values significantly different from solvent alone (p≤0.05 as determined by the students T-test) are indicated by an asterisk. (TIF) [file pone.0056860.s002.tif]
